# Spatial Studies on Vector-Transmitted Diseases and Vectors

**DOI:** 10.1155/2012/573965

**Published:** 2012-11-20

**Authors:** Maria Goreti Rosa-Freitas, Nildimar Alves Honório, Cláudia Torres Codeço, Guilherme Loureiro Werneck, Nicolas Degallier

**Affiliations:** ^1^Laboratório de Transmissores de Hematozoários, Fundação Oswaldo Cruz, Avenida Brasil 4365 Manguinhos, 21045-900 Rio de Janeiro, RJ, Brazil; ^2^Programa de Computação Científica, Fundação Oswaldo Cruz, 21045-900 Rio de Janeiro, RJ, Brazil; ^3^Instituto de Medicina Social, Universidade do Estado do Rio de Janeiro, 20550-900 Rio de Janeiro, RJ, Brazil; ^4^IRD UMR182, Laboratoire d'Océanographie et du Climat, Expérimentation et Approches Numériques (LOCEAN) Tour 45-55, 4e étage, case 100, 4 place Jussieu, 75252 Paris Cedex 5, France

## 1. Space, the First Frontier

Space is the stage where factors leading to disease take place.

Landscape epidemiology, health geography, spatial epidemiology, and landscape ecology^1^ are disciplines that share a fundamental concept: space.

Space as herein defined is the multidimensional extent in which elements and historical events—geological, physiographical, ecological, climatic, economical, and cultural—concur and interact with humans to influence the presence, development, activity, and longevity of pathogens, reservoirs, and vectors, leading to different patterns of infection and disease.

Disease is a spatially-determined phenomenon [1–3]. The correct identification of spatial risk factors plays a key role in prediction, prevention, and control of disease [4–7].

The analysis of space and its role in diseases has been occupying thinkers since Hippocrates *circa* 400 B.C. Terms like tropical diseases, malaria, and American trypanosomiasis just to name a few, have geographically-oriented denominations that immediately refer to spaces where transmission occurs^2^.

According to Hippocrates, an endemic disease is determined by the nature of a certain place. The term endemic^3^ entails in itself this concept. Hippocrates' ecological concept of disease was brought up again by Galen in the early Christian era and passed untouched through the modernization of science during the Renaissance.

## 2. The Study of Space

Spatial analysis is concerned with the geographic space, that is, observations that correspond to locations in space that capture their proximity in the real world. The interrelation between entities increases with proximity in the real world and their representation in geographic space and assessment using spatial analysis techniques are appropriate (Tobler's first law of geography, [8]) and in accordance with the concept of spatial dependence that forms the foundation of spatial analysis.

In 1939, Pavlovsky structured the theory of the natural nidality^4^ of transmissible diseases, known outside the Soviet Union only by late 1950s [2, 3]. Pavlovsky's theory consisted of 3 axioms: (1) diseases tend to be limited geographically; (2) this spatial variation arises from underlying variation in the physical and/or biological conditions that support the pathogen and its vectors and reservoirs; (3) if those abiotic and biotic conditions can be delimited on maps, then both contemporaneous risk and future change in risk should be predictable [9].

A landmark on spatial analysis was Snow's 1854 map of cholera deaths and the water pumps that supplied the city. The superposition of cholera cases and a main water pump gave support to his hypothesis that cholera was spread by the water [10].

Spatial analysis comprises a set of generic exploration methods and the visualization of data in the form of maps.

Nonetheless, spatial analysis is neither a technology for making maps nor a map is the sole objective to be achieved. The interaction with mapped variables resulting from spatial analysis is more challenging than the interaction with traditional maps and spatial records. Spatial analysis has changed our perspective of viewing a map. It has moved mapping from a historical role of input provider to a dynamic and essential element in the decision-making process [11].

Spatial analysis exploration and visualization methods allow not only the visual description of the distribution of variables but also the identification of patterns in the spatial distribution making it easier to comprehend the phenomena underlying these observations. Through these procedures it is possible to select the most accommodating inferential model [12], to choose the best explanatory hypothesis and propose control scenarios supported by the spatial relationships observed.

## 3. Spatial Studies Tools

Visual inspection of spatial distribution of data allows apprehending existing patterns. The translation of these patterns into a system of theoretical significance is an important tool in the investigation of the disease process.

Currently, spatial studies of disease and vectors involve the use of computational analyses and representation of geographic data using the so-called geographic information systems-GIS or spatial analysis tools, as in the broader sense used in this text. These spatial analysis tools perform the computational treatment of georeferenced data points, lines, and areas and store their attributes in relation to the earth surface and represented in a cartographic projection [12]. Advances in remote sensing, global positioning systems, computer software, and hardware led to the creation of powerful data exploratory tools.

A spatial-related geographic database is composed of georeferenced data input and integration, graph, and image processing functions, visualization, and plotting, spatial analysis tools, data storage, and retrieval in an organized form.

After being subjected to visualization techniques, hypotheses on spatial behavior of data are challenged to validation and corroboration through spatial analysis and theoretical models.

Besides being able to create static risk maps based on distributions of vectors, reservoirs and disease incidence, spatial analysis can model spatiotemporal dynamics and show how the spatial distribution of infectious diseases changes through space and time. Where field data are unavailable, predictors of disease risk can be applied.

Spatial analysis is both quantitative and qualitative. Neither quantitative nor qualitative methods are end tools. Quantitative spatial analyses with predictive models and qualitative scrutiny and theories help to gain a better understanding of visualized data and the underlying processes within.

Nature of spatial data affects the type of spatial analysis to be employed and interpretation.

Data selection, data cleaning, and preprocessing and, moreover interpretation of results are all part of the subjective qualitative interpretation. Subjective analysis should be ascertained as an important tool. The testing of different analyses should be extensively used to aggregate knowledge on the problem. Spatial analysis results should be used to corroborate or reject findings and applied to mathematical models for interpretation and theory construction. These in turn, will be used for building up knowledge on disease dynamics and ultimately for practical control issues like policy and management decisions and, for checking particular interventions.

## 4. In This Issue

The papers comprised in this special issue contain up-to-date methods of spatial analyses applied to the study of diseases and vectors. These papers investigate spatial and temporal scales, and develop risk maps and models aiming to increase our understanding as well as supporting decisions in control programs.

In order to reduce malaria transmission in Zambia, a spatial study was developed to guide the implementation of effective vector control measures, and increase the understanding of the interactions between epidemiological and entomological malaria transmission determinants and the impact of interventions (E. Chanda et al. in this special issue). Monitoring the impact of vector control through a spatial-based decision support system revealed spatial variations in the prevalence of infection and vectors which are susceptible to insecticides. It also enabled the characterization of the spatial heterogeneity and the identification of areas with reduced parasitaemia and increased insecticide resistance. The spatial-based decision support system proposed by E. Chanda et al. provided opportunity for rational policy formulation and cost effective utilization of limited resources for enhanced malaria vector control.

Major spatial changes induced by man may lead to disequilibrium that in turn may result in human disease. This is the hypothesis raised by Paula et al. (in this special issue). The closure of a dam in São Paulo, Brazil, favoured the proliferation of aquatic weeds, the main habitat of *Mansonia *mosquito species (M. B. de Paula et al. in this special issue). This event led to a dramatic increase of the *Mansonia humeralis *population, from 3 to >50%. *Ma. humeralis *is a persistent biter provoking nuisance in the human population and potentially facilitates the transmission of arboviruses. A spatial-oriented sustainable entomological control was advised for this area.

Although Brazil was declared free from Chagas disease transmission by the domestic vector *Triatoma infestans *by the World Health Organization in 2006, vector-transmitted human acute cases are still being registered (R. Gurgel-Gonçalves et al. in this special issue). In order to assess Chagas disease transmission risk, distribution models for 62 Brazilian triatomine species were generated (R. Gurgel-Gonçalves et al. in this special issue). Although most actual occurrences were recorded in open areas of the Brazilian savannah (*cerrado* and *caatinga*), spatial analyses and distribution models showed that Brazil is, as its most, vulnerable to Chagas vector-borne transmission.

Spatial analysis was applied in a study of *Biomphalaria *snail species, which are the intermediate hosts of *Schistosoma mansoni* in Minas Gerais, Brazil, to optimize resource allocation (R. J. P. S. Guimarães et al. in this special issue). Kriging showed to be a rather robust tool since its results presented a good agreement with the field findings. This tool allowed the delimitation of the *Biomphalaria* distribution, characterizing the uncertainty of areas at risk.

Buruli ulcer is a debilitating human skin disease with an unknown transmission mode with epidemiological data linking it to swampy areas. Data available suggest that aquatic insects play a role in the dissemination and/or transmission of this disease. However, aquatic insect biodiversity and biology in Africa remain poorly documented. Entomological survey in Bankim, Cameroon, an area recently described as endemic for Buruli ulcer was conducted in order to identify the commonly occurring aquatic bugs and document their relative abundance, diversity, and spatial distribution (S. M. A. Ebong et al. in this special issue). Abundance, distribution and diversity of aquatic bugs varied according to type of aquatic environments and maybe used for future risk maps assessment.

Control actions for visceral leishmaniasis in Minas Gerais, Brazil, showed that the use of an automated database with geoprocessing was important to guide control measures (L. Saraiva et al. in this special issue). In fact, the use of spatial analysis tools promoted greater efficiency in the decision making and planning activities especially for urban areas where many of the disease characteristics are unknown.

Spatial-temporal analysis of the abundance of phlebotomines vectors of tegumentary and visceral leishmaniasis was performed in Argentina compared spatio-temporal scales (M. G. Quintana et al. in this special issue). Microscale, mesoscale, and macroscale analyses resulted in different spatial observations. These observations raised the awareness of scale choice and consistency in conclusions from spatial studies. Scales from microfocal to regional, although within each other in increasing order, require questions, resolution, data quality, and different analytical tools, to support the conclusions appropriate to each scale.

## 5. Space in the Future

Simple interactions among proximal entities can lead to complex, persistent, and functional spatial entities at higher aggregate levels. Spatial studies of diseases and vectors seem to be an excellent platform from which to explore these issues.

Spatial data comes in many varieties and it is not easy to arrive at a system of classification that is simultaneously exclusive, exhaustive, imaginative, and satisfying [13].

Innovative research on spatial analysis has been building newer insights into fundamental issues as structure of theories, models, technologies, and new methods of representation that go beyond earlier GIS models, giving birth to new techniques for addressing uncertainty [14].

The increasing ability to capture and handle geographic data means that spatial analysis is occurring within increasingly data-rich and growing analytical power environments. This wealth of new processing capabilities provides an opportunity to address complex spatial issues in entirely new ways [11].

The use of spatial models to generate potential distribution and risk maps, followed by careful assessment of models, could lead to increment in knowledge of the different properties of a system at different levels of aggregation and in different study fields to target interventions to prevent, manage, and control disease [9].

In the search for valid and reliable conclusions a variety of exploratory and confirmatory, qualitative and quantitative procedures are being developed daily.

The incorporation of spatial analytical functionality within commercial and open-source GIS, the linkage of specialized statistical and other analytical modules have attained a conspicuous progress in spatial studies [15]. 

Nonetheless, the pursuit for methods and tools that allow more specific and ever demanding treatment of space in empirical applications in many sciences, in measurement, in stressing space-time dynamics, in hypothesis development and in validation of theoretical constructs will never cease to exist. This pursuit was valid a decade ago [15] and still is today.

We have started witnessing new map forms and processing environments. 

New maps forms go beyond the 2D paper map. Users often require being able to have spatial information on a 3D view of the terrain. Virtual reality can transform the information from polygons to objects near photographic realism [11]. A four dimension spatial analysis tool system (3D plus time) is currently the major challenge. Currently, visualization of time is only possible by animation of a series of map layers. 

Among the new processing environment are full integration of the global positioning system and remote sensing imagery with spatial analysis in real-time applications. 

Typical commercial GIS toolbox software products are being substituted by web spatial analysis services where users customize their own views. 

High-level applications have been initiated by the free release of data in the web and the ability of using analytical tools and display capabilities from different sites resulting in complex data manipulation which however does not require specific GIS knowledge from the end user. In this new environment, the user focuses on the spatial logic of a solution and is hardly aware that spatial analysis is even involved [11]. 

A combination of spatial studies innovations derived from other sciences and interdisciplinary expertise might lead to advancements farfetched from currently used tools and observed outcomes. These spatial studies will most likely take us closer and closer to unveil the natural complexity of components of disease in space. 

*Maria Goreti Rosa-Freitas* *Maria Goreti Rosa-Freitas* *Nildimar Alves Honório* *Nildimar Alves Honório* *Cláudia Torres Codeço* *Cláudia Torres Codeço* *Guilherme Loureiro Werneck* *Guilherme Loureiro Werneck* *Nicolas Degallier* *Nicolas Degallier*

## Endnotes

1.
*Ecology.* (Greek *oikos* = house, dwelling place, habitation; *logia* = study of) The study of the relationships between living organisms and their environment. The term *Ökologie* was coined in 1866 by the German scientist Ernst Haeckel (1834–1919) [16]. *Landscape.* A portion of land or territory which the eye can comprehend in a single view, including all the objects it contains [17]. A heterogeneous land area composed of a cluster of interacting ecosystems that is repeated in similar form throughout [18]; an area of land containing a mosaic of habitat patches, often within which a particular focal habitat patch embedded [19] relevant to the phenomenon under consideration at any scale where each species is likely to respond to more than one scale [20]. Originally introduced as a painters' term c. 1600 as a painting representing natural scenery, old English *landscipe*, Germany *landschaft*, old Norwegian *landskapr*; the meaning tract of land with its distinguishing characteristics is from 1886 [16]. *Geography.* (Greek *geo* = earth, *graphia* describe-write) The study of lands, features, inhabitants and phenomena of Earth. First used by Eratosthenes (276–194 bC) [21]. *Habitat.* Latin = it inhabits. An ecological or environmental area that is inhabited by a particular organism. The natural environment in which an organism lives, or the physical environment that surrounds (influences and is utilized by) a species population [22]. As a technical term in Latin texts on English flora and fauna, literally it inhabits in 1762. From the verb *habitare* = to live, dwell. General sense of dwelling place is first attested in 1854 [16]. *Environment.* The circumstances, objects, or conditions by which one is surrounded. The complex of physical, chemical, and biotic factors (as climate, soil, and living things) that act upon an organism or an ecological community and ultimately determine its form and survival. The aggregate of social and cultural conditions that influence the life of an individual or community [23]. State of being environed or surrounded c. 1600. Nature, conditions in which a person or thing lives, first recorded in 1827, used by Carlyle to render German *Umgebung*. Specialized ecology sense first recorded in 1956 [16].

2.
*Malaria.* Italian. mal'aria, from mala aria, literally bad air, from *mala* = bad (feminine of *malo*, from Latin *malus*) + *aria* = air. Probably first used by Italian physician Francisco Torti in 1740 (1658–1741). The mosquito-borne disease was once thought to have been caused by foul air in marshy districts [16]. *American Trypanosomiasis.* An anthropozoonosis due to the protozoan *Trypanosoma cruzi* transmitted to man by infected faeces of a blood-sucking triatomine bug, skin break or through mucous membranes, contaminated food, blood transfusion, pregnancy and delivery, organ transplantation or laboratory accident endemic of Central and South Americas [24].

3.
*Endemic. ε* *νδ* *ημ* *ία* *en* in, within, inside + *demos* people or population and also place, home.

4.
*Nidality.* Nidus according to Pavlovsky, a translation of Russian *ochag* = hearth, breeding ground. Thus a nidus of a disease is its nest, home, or habitat, equivalent to the Latin focus [25].
